# Hereditary hemorrhagic telangiectasia, liver disease and elevated serum testosterone (Osler–Weber–Rendu syndrome): a case report

**DOI:** 10.1186/s13104-017-2397-z

**Published:** 2017-01-23

**Authors:** R. Dissanayake, K. P. K. Y. M. D. S. Wickramarathne, S. N. Seneviratne, S. N. Perera, M. U. J. Fernando, V. P. Wickramasinghe

**Affiliations:** 1 Department of Paediatrics, Faculty of Medicine, University of Colombo, Colombo, Sri Lanka; 2 Lady Ridgeway Hospital for Children, Colombo, Sri Lanka

**Keywords:** Hereditary hemorrhagic telangiectasia, Arterio venous malformation, Liver nodules, Elevated serum testosterone

## Abstract

**Background:**

A Sri Lankan girl with hereditary hemorrhagic telangiectasia (Osler–Weber–Rendu syndrome) is described.

**Case presentation:**

She presented with recurrent spontaneous epistaxis, pulmonary arterio venous malformation and oral telangiectasia. A diagnosis of Hereditary hemorrhagic telangiectasia (Osler–Weber–Rendu syndrome) was made based on the presence of three Curacao criteria (out of four). Evaluations of her jaundice revealed chronic parenchymal liver disease with multiple nodules in the liver with early portal hypertension. She had a muscular build, with elevated serum testosterone but low serum dehydroepiandrosterone sulphate levels. This could be attributed to impaired sulfation of dehydroepiandrosterone due to portocaval shunting of blood, leading to hyperandrogenemia.

**Conclusions:**

Hyperandorogenemia due impaired sulfation of dehydroepiandrosterone as a result of portocaval shunting is seen in Hereditary haemorrhagic telangiectasia.

## Case description

### Background

A 16-year old girl from a remote village in Sri Lanka is described.

### Case presentation

She was referred from the Paediatric Cardiology Unit to the University Unit of Lady Ridgeway Hospital for Children (LRH) for further evaluation of jaundice. She was the only child born to non consanguineous healthy parents and was well till 14 years of age, when she sought medical advice from the local hospital for exertional dyspnoea. At presentation to the local hospital she was detected to have central cyanosis and clubbing but otherwise clinically normal. Her haemoglobin was 18 g/dl and the packed cell volume was 54%. Her platelet count was 73 × 10^9^/l and SpO_2_ 82%. The echocardiogramme revealed a pulmonary arteriovenous malformation (AVM). The ultra sound scan (USS) of the abdomen showed a normal liver and no splenomegaly.

She was referred to the cardiology unit of LRH for further evaluation. At LRH contrast echocardiogramme was performed which showed evidence of pulmonary arteriovenous fistula. The finding at cardiac catheterization was compatible with bilateral diffuse pulmonary AVMs. Due to the diffuse nature of the pulmonary AVM she was managed conservatively.

Two years later, at the age of 16 years she developed spontaneous recurrent epistaxis. She was also noted to have jaundice. Examination revealed deep jaundice, plethora, central cyanosis and clubbing. There were no other peripheral stigmata of chronic liver disease. She had coarse facial appearance. There was no cutaneous telangiectasia. Abdominal examination revealed a firm hepatomegaly of two cm, but no splenomegaly or ascites. Rest of the abdominal examination was normal. Cardiovascular system examination revealed a systolic murmur best heard over the pulmonary area. Blood pressure was normal. Rest of the examination including respiratory system and central nervous systems were clinically normal. Ultra sound scan examination of abdomen at sixteen years revealed heterogeneous linear (Liver) echo texture, increased peri portal echogenicity with splenomegaly and varices compatible with chronic liver disease and early portal hypertension. A focal lesion was seen in segment Vl of the liver. Magnetic resonance imaging (MRI) revealed a dilated splenic vein draining separately through the diaphragmatic hiatus between the inferior vena cava and the aorta possibly into the superior vena cava through the azygous vein. She continued to be managed conservatively.

At nineteen years of age she remained jaundiced. There were telangiectases over the oral mucosa and conjunctiva. Eye referral revealed telangiectatic vessels in the right optic disc. Hepatomegaly was unchanged.

She had attained menarche at 16 year of age but had amenorrhea for several years, and now has her menstrual periods infrequently. Although she has a masculine habitus her secondary sexual characteristics were feminine. There was no clitoromegaly. Endocrinological evaluation was done to investigate for her masculine built and irregular menstruation. There was no family history suggestive of polycystic ovarian syndrome. At 19 years of age, she had an elevated serum testosterone level of 15.97 nmol/L (normal adult female range 0.52–2.43), but serum DHEAS (dehydroepiandrosterone sulfate) level were low (1.0 μmol/L, normal range 1.7–13.4). Serum 17 OHP, FSH, LH and serum estradiol levels were normal. She also had marginally low free T4, with normal TSH. USS abdomen repeated at 19 years of age was compatible with chronic liver disease with early portal hypertension. She had normal uterus and normal ovaries with no evidence of poly cystic ovaries.

**Fig. 1:**
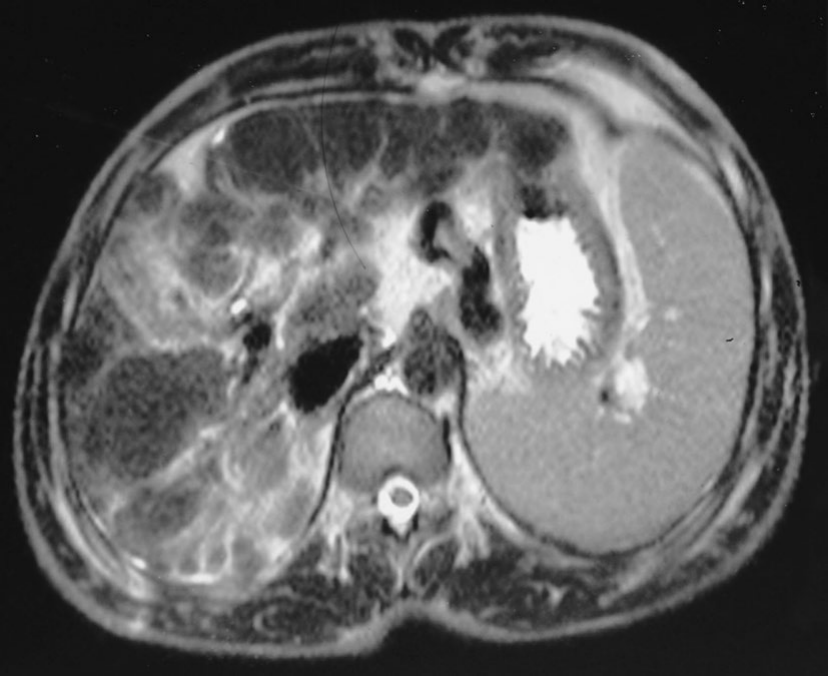
Magnetic resonance imaging showing multiple nodules in the liver

MRI abdomen (Fig. 1) showed changes of chronic parenchymal liver disease with multiple nodules in both lobes of the liver. The nodules were hyperplastic with low T2 signal intensity. A 47 × 45 mm size nodule was seen in the right lobe of the liver. A liver biopsy was not done due to the risk of bleeding in the presence of multiple AVMs. MRI brain done to identify any cerebral vascular malformation was normal.

A diagnosis of hereditary hemorrhagic telangiectasia (Osler–Weber–Rendu syndrome) was made based on the presence of three out of four Curacao criteria [1]. The three criteria she fulfilled were recurrent spontaneous epistaxis, visceral (pulmonary) arterio venous malformation and telangiectasia in the oral mucosa [1]. The patient along with the family was counselled regarding the diagnosis and the prognosis.

## Conclusion

This 19 year old girl has Hereditary haemorrhagic telangiectasia with chronic liver disease and pulmonary AVM. She has a masculine appearance due to impaired sulfation of dehydroepiandrosterone (DHEA) as a result of portocaval shunting.

Hereditary hemorrhagic telangiectasia—HHT (Osler–Weber–Rendu syndrome) was first described in the nineteenth century as a familial disorder causing nose bleeds, gastrointestinal bleeding and abnormal vascular structures. It is inherited as an autosomal dominant trait. A diagnosis of HHT could be made based on Curacao’s criteria, which are; Spontaneous recurrent epistaxis, multiple telangiectases at characteristic sites such as lips, oral cavity, fingers, nose, visceral lesions such as gastrointestinal telangiectasia, pulmonary AVM, hepatic AVM, Cerebral AVM and spinal AVM, a family history of a first degree relative with HHT. The presence of three criteria makes it a definite diagnosis of HHT, while two make the diagnosis possible. Our patient had spontaneous recurrent epistaxis, visceral AVM in the form of pulmonary AVM and telangiectasia in the oral mucosa. There was no family history of HHT. Based on these three criteria a definitive diagnosis of HHT was made [1]. The diagnosis was made at 19 years of age as the telangiectasia of the oral mucosa appeared only at nineteen years.

Spontaneous recurrent nose bleeds from telangiectasia of the nasal mucosa are the most common manifestations of HHT and occurs in about 95% [2]. Intervention for epistaxis should be considered if there is anaemia attributable to nose bleeding or if the frequency and duration of bleeding interferes with normal activities [2]. Our patient required no treatment as the epistaxis was self-limiting. Telangiectases of the skin and buccal mucosa also occur in about 95% of patients, typically presenting later in life than epistaxis [2]. It usually presents in about the third decade of life and gradually increases in number and size. They mostly occur in the face, lips, tongue, buccal mucosa and finger tips and can occur elsewhere. Our patient had telangiectases over the oral mucosa and conjunctivae.

Pulmonary AVMs are thin walled abnormal vessels that replace normal capillaries between pulmonary arterial and venous circulation. In one study 37% of patients with HHT demonstrated pulmonary AVM on high resolution computed tomography (CT). The safest method for treatment is transcatheter embolisation. Since our patient had bilateral diffuse pulmonary AVM conservative management was planned. Cerebral AVMs occur in approximately 10% of patients with HHT [2]. They can cause headache, seizures, ischaemia of the surrounding tissue, or haemorrhage. Our patient did not have cerebral involvement.

Hepatic involvement is variable in HHT. In one study only 8% showed symptomatic involvement [2]. In HHT, the predominant liver involvement seen was shunting of blood from hepatic artery to hepatic veins with shunting of metabolites from portal circulation to systemic circulation. A shunt from hepatic artery to portal vein leads to portal hypertension and increased blood flow can lead to nodularity of the liver due to increased deposition of fibrous tissue. The liver undergoes nodular transformation also known as “pseudo-cirrhosis” [3]. In our patient there was no vascular malformation of the liver however nodular hyperplasia was seen. She did not undergo liver biopsy as there is a risk of bleeding. Liver transplantation is currently the only curative option for symptomatic liver disease in HHT. Except for hepatocellular and biliary necrosis which requires urgent liver transplantation consensus on the most appropriate timing of the transplantation is lacking [4].

To our knowledge, elevated serum testosterone levels have not previously been reported in females with HHT. However, there have been a few reported cases of elevated serum testosterone in association with porto-systemic shunting in young females [5, 6]. The initial observation was reported in two adolescent girls with congenital porto-systemic shunting, who presented with virilisation and amenorrhea, and had elevated testosterone [5]. More recently, Bas et al. [6] reported on two 7-year old girls with congenital portosystemic shunts, presenting with premature pubarche. These females had elevated serum testosterone and other downstream androgens in the presence of low DHEAS, as seen in our patient [6]. It is postulated that the mechanism of hyperandrogenemia, could be impaired conversion of DHEA to its inactive sulphate ester DHEAS in the liver, due to bypassing of DHEA to the systemic circulation [6]. This would lead to low DHEAS and elevated DHEA in the circulation. As DHEA is a precursor for the synthesis of more potent downstream androgens such as androstenedione and testosterone, elevated DHEA would lead to elevated serum testosterone and features of hyperandrogenism [6].

This is a classical case of HHT with hyperandrogenaemic features. This probably could be the first reported case with hyperandrogenaemia secondary to hepatic AVM leading to shunting of blood leading to underclearance of proandrogenic metabolites from the circulation.

## Abbreviations

LRH: Lady Ridgeway Hospital for Children
AVM: arteriovenous malformation
USS: ultra sound scan
DHEAS: dehydroepiandrosterone sulfate
DHEA: dehydroepiandrosterone
HHT: hereditary hemorrhagic telangiectasia
MRI: magnetic resonance imaging
